# Redefining cognitive neurodynamics through transdisciplinary innovation

**DOI:** 10.1007/s11571-025-10332-z

**Published:** 2025-09-05

**Authors:** Alessandro E. P. Villa

**Affiliations:** NeuroHeuristic Research Group, University of Lausanne, UNIL Chamberonne Internef 138.1, 1015 Lausanne, VD Switzerland

**Keywords:** Neuroheuristics, Neural networks, Cognitive dynamics, Dynamical systems, Neuromodulation, Deterministic chaos

## Abstract

This paper introduces the concept of *neuroheuristics*—a novel transdisciplinary paradigm designed to advance cognitive neurodynamics by integrating insights from molecular biology, computing, behavioral science, and clinical neuroscience. Contrasted with the traditional reductionist approach rooted in classical determinism, neuroheuristics emphasizes a flexible, problem-solving methodology for investigating brain function across multiple levels of complexity. The paper explores the epistemological interplay among genetic, epigenetic, and environmental factors in brain development and pathology. The neuroheuristic framework aims to elucidate complex cognitive phenomena-such as memory, decision-making, and creativity-by bridging bottom-up and top-down research strategies. By incorporating contemporary technologies and recognizing the brain’s dynamic, nonlinear properties, neuroheuristics proposes a transformative shift in cognitive neurodynamics, enabling a deeper understanding of human cognition, disease mechanisms, and artificial intelligence. Its applicability is demonstrated through ongoing interdisciplinary research spanning neurophysiological disorders, computational modeling, and data-driven analytical techniques.

## Introduction

Modern science is grounded in the assumption of an objective external reality, a perspective historically reinforced by the rational and mechanistic principles of causality and determinism. This philosophical stance, which originated with Descartes in the 17th century, has been fundamental to the development of classical empiricism. It has profoundly influenced progress in the physical and technical sciences, shaping the modern technological world we live in today. While these principles have led to major successes in the physical sciences, their limitations become evident in complex, adaptive systems-particularly in biology. In fluid dynamics, for example, understanding turbulent flow has necessitated the incorporation of chaos theory, which departs from classical deterministic frameworks (Frisch 1995; Pope 2000). Similarly, in biomedical research, the limits of classical approaches become evident, especially when studying aging and pathological processes. The apparent stability of living organisms is deceptive, as most intracellular molecules are constantly being renewed. This continuous cycle of anabolism and catabolism, the breakdown and rebuilding of cellular components, is a major consumer of energy in organisms. These transformations are not adequately explained by classical thermodynamics alone and are more accurately described through the framework of far-from-equilibrium thermodynamics, where energy dissipation facilitates transitions toward more stable organizational states (Prigogine and Stengers 1979).

Such reorganizations involve complex flows of information that resist quantification through traditional spatial metrics. When applied to brain function, this epistemological challenge becomes particularly pronounced. Cognition cannot be isolated from its neurobiological substrate, nor can it be fully explained without reference to mental representations, logic, and computational models. Biological systems are subject to both genetic and epigenetic variability, which can result in significant cellular disruptions during development. For instance, up to 20-50% of neurons undergo programmed cell death (*apoptosis*) after they have matured and established functional connections (Andersen 2004; Dekkers et al. 2013; Riva et al. 2019). Though counterintuitive from the standpoint of classical developmental biology, this pruning process is essential for enabling higher-order neural organization.

It remains problematic to assert that mental phenomena-particularly those shaped by individual experience-can be directly reduced to cellular or molecular levels of neural organization. Historical examples underscore this complexity. For instance, the brain of Anatole France, a Nobel Prize-winning writer (France 1921), was comparable in weight to that of *Homo erectus* (Java Man) (Keith 1927), and the Neanderthal brain was, in fact, larger than that of modern humans (*Homo sapiens*) (Falk 1991; Tattersall 2023). Such examples highlight that brain volume alone is insufficient to explain cognitive capacity. The functional architecture of the brain emerges from multiscale interactions that span molecular, cellular, individual, and social dimensions (Koch 2016; DeSilva et al. 2021).

The brain’s functional complexity—operating across these multiple organizational scales-poses a major challenge to any unified explanatory model of cognition. It is within this conceptual and methodological gap that the neuroheuristic paradigm is proposed: as a framework capable of addressing both the nonlinear, dynamic nature of brain function and the epistemological limitations of reductionist science.

## From complexity to discovery: a new paradigm for neuroscience

In neuroscience, researchers have traditionally adopted a “bottom-up” approach, beginning with cellular or molecular processes and working upward toward the understanding of complex cognitive functions. Although widely employed in neuroscience, this reductionist strategy remains constrained by experimental limitations. Even the most basic neural architectures are too complex to be exhaustively characterized at the cellular level. Conversely, “top-down” methodologies—those that start with behavioral or cognitive phenomena and attempt to infer underlying mechanisms—can offer more practical heuristics for system-level modeling, yet often lack the mechanistic specificity needed to explain neural dynamics in detail. While each approach yields valuable insights, neither is sufficient in isolation. The convergence of “bottom-up” and “top-down” strategies holds transformative potential: their integration is not merely additive but generative, fostering the development of new conceptual frameworks and leading to unexpected discoveries. In the 21^st^ century, this integrative approach must continuously incorporate emerging sciences and technologies. Two areas that are particularly crucial today are molecular biology and computer science.

The interdisciplinary convergence provided by their methodological and analytical tools drives the development of a truly transdisciplinary theoretical framework (Kötter and Balsiger 1999) in integrative neuroscience—a shift exemplified by the emergence of the *neuroheuristic* paradigm. The term *neuroheuristics* (alternatively, *neuristic*) derives from the Greek words νεῦρον [*neuron*], meaning ‘nerve’, and εὑρίσκειν [*heuriskein*], meaning ‘to find’ or ‘to discover’). It denotes a transdisciplinary epistemology that transcends the boundaries of specialized expertise, promoting iterative knowledge renewal as a fundamental feature of scientific inquiry. In this framework, outcomes are not easily classifiable as successes or failures, because progress is measured by the continual refinement and transformation of explanatory models. The set of procedures or frameworks that define how we come to know something, and how we determine that our knowledge is credible or meaningful (i.e., the epistemologic process) cannot be reduced to mere proficiency. The emphasis lies not in static expertise, but in adaptive methodologies capable of responding to the brain’s inherent dynamism. This stands in contrast to Henri Bergson’s (1859-1941) concept of psychophysical interactionism, Bergson (1896), wherein progression is driven by a “vital impulse” (*élan vital*)—a metaphysical force responsible for evolutionary transitions (Bergson 1907). Whereas Bergson attributes change to a life-force beyond physical properties, neuroheuristics conceptualizes transformation as an emergent property of complex interactions among existing system components.

One of the central aims in neuroheuristics is to explain how the brain integrates information across hierarchical levels of organization—from molecular interactions and neuronal circuits to cognitive processes and behavior. Recent scientific and technological developments, especially in genomics and computational neuroscience, offer unprecedented tools for probing such multilevel interactions and driving new insights and discoveries. For instance, the Human Genome Project (1990–2003) dramatically accelerated the understanding of genetic contributions to brain structure and function. Approximately 40% of human genes are expressed in the brain (Wang et al. 2018; Sjöstedt et al. 2020), and hundreds of heritable neurological disorders have been identified (Cooper and Jan 1999; Shukla et al. 2021). Although there are an estimated 20,000 to 25,000 genes that encode proteins, not all genes are constantly expressed in every cell. It is now widely recognized that gene expression is highly dynamic—modulated by cell type, developmental stage, and environmental stimuli. Furthermore, non-coding regions of the genome, once dismissed as “junk DNA”, play critical roles in gene regulation. The etiology of many neurological and psychiatric disorders involves complex interactions between polygenic risk factors and environmental exposures, presenting an analytical challenge that resists simplistic models of causality.

Historically, numerous scientific breakthroughs have emerged from the study of rare diseases and small populations. For example, Brown and Goldstein’s Nobel Prize-winning research on cholesterol metabolism focused on a rare genetic disorder characterized by extremely high cholesterol levels (Brown 1985; Goldstein 1985). Their findings ultimately illuminated mechanisms relevant to the broader population suffering from hypercholesterolemia. These cases illustrate the scientific value of studying atypical phenomena-an approach aligned with heuristic reasoning.

Unlike algorithmic methods, which guarantee correctness but often require impractically long computation, heuristic strategies prioritize adaptability and iterative progress. Though they do not ensure optimal solutions, heuristics enable the generation of satisfactory outcomes within well-defined constraints. In clinical medicine, for example, heuristic tools such as decision rules allow physicians to revise diagnoses in real time based on emerging patient data, fostering faster and more adaptive care (Guy et al. 2016). In neuroscience, Adaptive Resonance Theory (ART) models learning as a continuous process of hypothesis evaluation and revision, enabling neural networks to adapt flexibly to novel sensory inputs and changing environments (Grossberg 1987, 1999). Similarly, in neuroimaging, initial heuristic interpretations of brain activity often guide the design of follow-up experiments, leading to improved analytical tools and deeper insights into brain–behavior relationships (Poldrack et al. 2017; Masulli et al. 2020; Perrig et al. 2010; Michel et al. 2012; Loosen et al. 2024). Studies on hierarchical processing in perception and cognition have also shed light on how complex neural circuits contribute to unified representations and adaptive behavior (Grossberg and Mingolla 1985; Grossberg 1999).

These diverse research efforts emphasize the integration of information across multiple levels of organization-a core principle of the neuroheuristic framework. This approach provides a pathway for understanding both the fundamental biological substrates of brain function and the emergent properties underlying cognition and disease. A practical example is found in Alzheimer’s disease research, where genetic markers, molecular pathologies, and network-level disruptions are examined in tandem to explain both biochemical mechanisms and cognitive decline (Celesia et al. 1993; Iturria-Medina et al. 2016; Hampel et al. 2018; Jack et al. 2018). Similarly, studies on attention-deficit/hyperactivity disorder (ADHD) integrate genetic vulnerabilities, neurotransmitter dynamics, and behavioral assessments to inform diagnosis and treatment across biological and clinical domains (Bonvicini et al. 2016; Mesrobian et al. 2018; Dotare et al. 2020; Cortese et al. 2022; Custodio et al. 2023). Neuromimetic computational models simulating these multilayered processes further provide experimental frameworks for exploring the complex causal pathways involved in neurological and psychiatric disorders (Villa 2008; Perrig et al. 2010).

## The neuroheuristic approach in cognitive neurodynamics

Cognitive neurodynamics refers to the study of how cognitive processes—such as perception, memory, decision-making processes, and attention—are dynamically organized and processed in the brain. It focuses on the temporal and spatial patterns of neural activity that give rise to cognition. The brain integrates both genetic predispositions (nature) and environmental influences (nurture) in processing information. This classical dichotomy traces back to early philosophical debates, with thinkers such as John Locke advocating the *tabula rasa* (blank slate) hypothesis (Wood 1992), in which individuals are shaped entirely by experience. Conversely, Charles Darwin emphasized the role of innate biological traits through his theory of evolution (Boero 2015). Contemporary science recognizes that nature and nurture are fundamentally intertwined-genetic predispositions are modulated, enhanced, or attenuated by environmental factors. For instance, an individual with a genetic inclination for musical ability may never realize their potential without access to appropriate environmental stimuli, such as musical education.

Within this dynamic interplay, neuroheuristics aims to maintain epistemological balance by emphasizing the relationships that govern brain development and function. A key question arises: do underlying laws govern this dynamic equilibrium? One of the earliest biological laws—Mendel’s laws of inheritance—provided the foundation for modern genetics (Keynes and Cox 2008). However, these laws apply within limited contexts and are insufficient to explain the behavior of living systems, which operate far from equilibrium. Classical mechanistic approaches, for instance, often treat time as a symmetric variable, permitting reversibility. Yet living systems are fundamentally governed by the irreversible flow of time. This concept, central to the work of Ilya Prigogine, emphasizes that classical physics is appropriate primarily for stable systems, while biological organisms require alternative principles grounded in far-from-equilibrium dynamics (Prigogine 1977; Prigogine and Stengers 1979).

Neuroheuristics aligns with this perspective, suggesting that the organization of the nervous system is fundamentally geared toward temporal information processing, such as memory and the ability to project oneself into the future—a function that is operationally defined as prediction. The prefrontal cortex plays a critical role in mediating these temporal contingencies and has been pivotal in the evolutionary process of hominization (Fuster 2015). Research into the temporal dynamics of neural activity has demonstrated that cognitive functions such as decision-making and reward processing rely on the intricate timing of neural circuits (Richmond et al. 2003; Abeles 2004; Bouret and Richmond 2010; Abeles 2014). One notable observation is that key processes, such as the interplay between memory and prediction, occur largely during sleep (Gais and Born 2004; Klinzing et al. 2019). Studies have shown that sleep, particularly its deeper stages, consolidates memories and facilitates the brain’s ability to make predictions based on past experiences. Disruptions in sleep (Horne 1978; Irish et al. 2012) can lead to severe memory impairments and an inability to access “future memories”—the set of action plans based on previous learning that guide present decision-making. Sleep deprivation, therefore, can lead to behaviors such as apathy, lack of foresight, and poor decision-making (van der Helm et al. 2010; Walker 2009; Coles et al. 2015). Another intriguing phenomenon observed during sleep is the absence of chronological references, which allows memories to blend with imagined or impossible scenarios-similar to virtual reality (Beattie et al. 2015; Picard-Deland et al. 2021). This process points to a temporal dimension of brain activity that differs from classical mechanics and may explain the sudden emergence of insights, ideas, or intuitions, sometimes referred to as the “Gestalt switch” (Wright 1992; Wray 2021; Metzinger 2024). These moments involve breaking free from conventional time constraints and are central to understanding creativity and innovation (Fell 2012).

Biological laws, much like mathematical axioms, were initially derived from observations and, while mostly correct, were limited by their descriptive nature. In contrast, neuroheuristics moves beyond this limitation by incorporating a broader, more flexible understanding of brain dynamics (Taylor and Villa 2001). Information flows through neural circuits at varying speeds (Stein et al. 2005; Rama et al. 2018), causing temporal distortions that are not well-described by classical spatial or metric models (Villa 2000; Abeles 2004; Vardi et al. 2013; Cabessa and Villa 2018). This is analogous to different communication systems (e.g., internet, surface mail, bike riders, Alpine horns, or flag signaling) delivering the same message at various speeds, each with its own symbolic system and syntax. This metaphor raises a critical question: how does the brain simultaneously manage multiple information codes?

The brain’s capacity for parallel, multichannel information processing presents unique challenges to conventional models of cognition (Braitenberg 1977; Palm 1982; Abeles 1991; Amit 1992). Neuroheuristics addresses this complexity by emphasizing continuous epistemological renewal at each stage of inquiry. This is especially relevant for understanding volition and decision-making processes that involve the integration of distributed information streams (Zhu 2004; Gold and Shadlen 2007; Haggard 2008; Bouret et al. 2012). Unlike traditional cognitive models that often rest on static theoretical constructs, neuroheuristics anticipates that hypotheses will be revised or surpassed. While cognitive science has significantly advanced our understanding of brain function by distinguishing between declarative knowledge (“knowing what”) and procedural knowledge (“knowing how”) (Atkinson and Shiffrin 1968; Squire 2004; Ryle 2009), it has also tended to reduce cognition to symbolic or computational processes. While cognitive science often uses computational models to simulate aspects of thinking and behavior, these models are typically based on algorithmic logic, such as those employed in classical Turing machines. Neuroheuristics, by contrast, emphasizes a more flexible and biologically grounded approach. Rather than assuming the brain functions like a step-by-step computer program, neuroheuristics integrates concepts from systems theory, nonlinear dynamics, and probability (Tetko and Villa 1997; Villa 2008; Cabessa and Villa 2018). This allows researchers to better model how the brain produces unpredictable, adaptive, and emergent behaviors (Freeman 2000; Brooks 1991; Jordanous 2020; Davis et al. 2025).

Historically, such synergies have fueled major advances in neuroscience. In the 18^th^ century, Albrecht von Haller conducted foundational experiments in animal physiology that investigated the brain’s responsiveness to physical stimuli (von Haller 1755). His work marked a turning point in neurophysiological thought, demonstrating the value of integrating principles from physics, chemistry, and natural philosophy. Later, the introduction of galvanic currents into the nervous system by Luigi Galvani (Galvani 1797) opened up a new frontier in neurophysiology and led to the birth of electrophysiology (Guarnieri 2014). Galvani’s findings, together with those of Luigi Rolando (Rolando 1809), marked a pivotal shift in neuroscience-from passive anatomical observation to systematic experimental investigation of brain function. This transition contributed to a paradigmatic transformation in scientific thinking, laying the conceptual groundwork for the development of modern theories of brain organization and function (Abbott 1999; De Palma and Pareti 2011; Agnati et al. 2007).

## Neuroheuristics in the age of computing: bridging historical insights with modern neuroscience

The heuristic paradigm introduced by early neuroscientists such as Albrecht von Haller and Luigi Rolando was not widely adopted by 19^th^-century researchers, which may partly explain why neuroscience lagged behind disciplines like mathematics, physics, and chemistry during that era. Yet, with hindsight, their approaches appear remarkably prescient. Just as electricity transformed neuroscience approximately two centuries ago, contemporary computing is reshaping the field today. The exponential growth of computational power enables neuroscientists to explore questions about information flow and brain dynamics that were once unreachable. This transformation exemplifies the neuroheuristic principle: discovery is not merely an accumulation of data but the continual refinement of models that link theory, computation, and empirical observation.

Today, computing has become indispensable to neuroscience, offering continually evolving tools that enable the study of increasingly complex systems. Computing power has progressed at such a rapid pace that hardware more than a few years old is often considered obsolete (Moore 2000; Brynjolfsson and McAfee 2014). This acceleration has enabled the resolution of problems once deemed intractable. For instance, cryptographic algorithms that would take millennia to solve through brute-force methods can now be tackled using heuristic strategies that deliver viable solutions within feasible timeframes (Rivest et al. 1978; Korf 1985; Kearns and Vazirani 1994; Papadimitriou 1994). This evolution challenges traditional notions of computational intractability and underscores the transformative role of heuristics in modern science.

Paradoxically, this rapid progress coexists with the need to preserve foundational structures, such as programming languages and architectural standards—a tension that is, in fact, an asset for neuroheuristics. While many computational models of neural networks emphasize dynamic properties, it is equally critical to account for biophysical constraints, such as the energetic demands of synaptic transmission and plasticity (Wang and Zhang 2007; Wang et al. 2015; Zhu et al. 2019). The neuroheuristic paradigm thrives on this duality, using computational advances not as ends in themselves but as flexible instruments for exploring the multilevel dynamics of brain function (Grossberg 2013).

Within this framework, it is useful to distinguish between two forms of rationality: instrumental and epistemic. Instrumental rationality concerns optimizing behavior to achieve specific goals, whereas epistemic rationality focuses on forming beliefs grounded in the best available evidence (Stanovich 2009). Both forms play pivotal roles in neuroscience and in real-world decision making. For instance, in legal contexts, judges must reach verdicts based not on absolute certainty, but on probabilistic reasoning supported by evidence-achieving a state of moral, if not empirical, certainty (Lietzau 1999; Guérin 2015). A similar principle applies in neuroscience, where hypotheses must be continuously tested and revised based on available data, acknowledging that complete certainty is rarely attainable.

Motivation is another central factor in understanding cognitive processing. Neural mechanisms underlying decision making frequently involve mutual inhibitory networks that resolve conflicts between competing action plans (Frith and Happé 1994; Berridge 2004). Dysfunction in these systems can result in maladaptive choices and impairments in personal and social behavior. Such deficits often correlate with learning difficulties and information-processing inefficiencies. These findings contributed to the formulation of the Somatic Marker Hypothesis, which posits that emotional processes play a critical role in guiding decision-making, especially under conditions of uncertainty (Bechara et al. 1994; Bechara and Damasio 2005). Cognitive and affective models of brain function help elucidate how decisions are shaped in contexts ranging from judicial reasoning to personal moral choice (Altimus 2017; Kraft and Giordano 2017).

Neuroheuristics employs computational models to explore the dynamic interactions between brain regions involved in learning, perception, and adaptation. Stephen Grossberg’s work on neural dynamics and Adaptive Resonance Theory (ART) provides foundational insight into how the brain stabilizes learning in the presence of complex, evolving stimuli (Grossberg 1987). His models illustrate how neural systems maintain internal coherence while remaining receptive to novel information-a process that aligns closely with the neuroheuristic approach to cognitive adaptability.

Advancing our understanding of cognitive function requires the formulation of provisional hypotheses-models that, while not universally valid, retain explanatory power within specific contexts (Villa 2008). For instance, studies on sensorimotor integration and decision-making processes, particularly in the auditory domain, benefit from a convergence of data sources: clinical observations in patients with executive function disorders, animal models, and computational simulations. This integrative methodology was instrumental in the founding of the Laboratory of Neuroheuristics at the University of Lausanne in 1995 (Villa 1985). From its inception, the laboratory aimed to overcome disciplinary compartmentalization by bringing together expertise from neurobiology, pharmacology, anatomy, physics, computer science, and electronics-laying the foundation for a genuinely transdisciplinary approach to brain research.

## Exploring brain complexity: key research themes in neuroheuristics

Neuroheuristics encourages researchers to move beyond fixed objectives and provisional hypotheses, fostering an open-ended approach to scientific inquiry. A useful metaphor is that of a child exploring a garden—curiously approaching boundaries such as hedges, trellises, or barriers—not to reach a predefined destination, but to discover what lies beyond. This metaphor underscores the value of intellectual flexibility, suggesting that rigid adherence to predetermined goals may hinder rather than facilitate discovery (Taylor and Villa 2001; Farina and Villa 2023).

Although scientific tools have evolved dramatically since the Neolithic era, the fundamental relationship between discovery and the instruments that enable it has remained constant. In recent years, technological advancements-especially in computing-have opened new frontiers in neuroscience. Medical imaging, for example, has transformed the study of brain structure and function. Yet, the complexity of modern neuroscientific challenges demands far more than computational efficiency. Neuroheuristics seeks to redefine this relationship by establishing a paradigm that synthesizes advances in computing, neuroscience, and molecular biology. The brain operates across multiple levels of organization, each contributing uniquely to cognitive function. At the microscopic level, neurons communicate via electrical and chemical signaling, forming complex circuits that underlie macroscopic processes such as memory, attention, and decision making. Understanding how these layers interact is central to contemporary brain science. At the Laboratory of Neuroheuristics, one research direction focuses on developing neuromimetic models and artificial neural networks that integrate empirical findings across scales.

A wide range of single-neuron models has been developed, from phenomenological approaches that simulate action potential generation (e.g., the leaky integrate-and-fire model) to biophysically detailed models that describe ionic dynamics across membrane compartments (Hill and Villa 1997; Asai and Villa 2008, 2012). Because many such models resist analytical treatment, practical formulations often rely on describing membrane potential fluctuations driven by diffusive processes. Increasingly, artificial neurons are being modeled to replicate the computational and adaptive properties of their biological counterparts (Villa and Tetko 1997). These biologically inspired models-referred to as *neuromimes*-are especially relevant for investigating the temporal characteristics of neural coding (Villa 2000; Del Prete et al. 2004; Villa 2008). Within this framework, the spiking activity of neurons is analyzed through spike trains-sequences of action potentials recorded via electrophysiological techniques (Tetko and Villa 2001b; Iglesias et al. 2005; Villa et al. 2007; Iglesias and Villa 2010).

Neuronal networks function as complex systems governed by coupled, nonlinear dynamical interactions. In studying large-scale networks, researchers often treat firing rates as continuous variables and employ mean-field models-originally developed for spin-glass systems-to characterize collective behavior. Typically, synchronization between systems is defined as shared periodicity and, in some cases, phase alignment (Villa and Tetko 2010; Abe et al. 2024). In the brain, interregional communication is mediated by oscillatory activity that synchronizes neural processes. However, classical synchronization models are often insufficient to account for brain dynamics, particularly under conditions driven by non-periodic or stochastic signals (Cabessa and Villa 2014; Masulli and Villa 2015). Indeed, biological neural networks frequently exhibit forms of synchronization that include chaotic behavior (Malagarriga et al. 2017, 2019).

While the functional significance of chaos in neural systems remains a topic of ongoing investigation, growing evidence supports its critical role in higher cognitive functions such as memory consolidation, decision-making, and creative insight. Skarda and Freeman (1987) argued that the brain leverages chaos not as noise, but as an intrinsic mechanism for perceptual reorganization and adaptive flexibility. In their view, chaos enables the brain to escape rigid attractor states, facilitating transitions to new neural configurations that underlie novel interpretations and responses. Expanding on this, Tsuda (2001), theory of “chaotic itinerancy” describes how neural systems traverse a landscape of quasi-stable states, allowing for context-sensitive cognition and spontaneous shifts in mental state-phenomena closely linked to creativity and problem solving. Empirical findings further substantiate these theoretical perspectives: chaotic dynamics have been observed in the primate motor cortex during motor planning, suggesting that such complexity may support behavioral adaptability in dynamically changing environments (Villa et al. 1998). Additionally, the detection of deterministic chaos in EEG recordings has proven valuable for predicting seizure onset, underscoring its clinical relevance (Aksenova et al. 2007; Villa and Tetko 2010). Furthermore, computational models that incorporate chaotic features provide insights into how flexibility and variability emerge from seemingly stochastic neural activity (Asai et al. 2008; Malagarriga et al. 2017). Within the neuroheuristic framework, chaos is not merely a computational artifact but a functional principle that enables the brain’s dynamic negotiation between stability and novelty.

Temporal dynamics constitute another critical dimension of brain complexity. Neural processes are inherently time-dependent, shaped continuously by attention, perception, and memory (Villa et al. 1998, 1999b; Jaquerod et al. 2020). Neural plasticity-the capacity of the brain to reorganize itself-exemplifies this dynamic adaptability (Villa et al. 1999a; Shaposhnyk and Villa 2012; Lintas et al. 2021). One behavioral paradigm illustrating this involves learning to associate complex sounds with specific motor responses. Over time, such sensorimotor integration strengthens connections between auditory and motor systems (Carretta et al. 1999; Storozhuk et al. 2002; Farré-Castany et al. 2007; Jaquerod et al. 2024).

Cognitive flexibility—the ability to shift strategies or adapt to new contexts—relies heavily on neuromodulatory systems that regulate neurotransmitters such as dopamine (Villa and Lorenzana 1997; Storozhuk et al. 2004; Lintas et al. 2012), serotonin (Hajós et al. 1995, 2001), and acetylcholine (Villa et al. 1996, 2000). These neuromodulators play crucial roles in regulating mood, learning, attention, and decision making (Aston-Jones and Cohen 2005; Mesrobian et al. 2015; Lintas et al. 2017). Although the term neuroheuristics has yet to be standardized, it increasingly denotes approaches that incorporate neuroscientific principles—such as neuromodulation—into heuristic models of cognition (Doya 2002; Cools and Robbins 2004; de Berker et al. 2016; Shine 2019). The neurochemical systems underlying such models are enormously complex, involving multiple neuromodulators and diverse receptor subtypes that are often co-expressed within individual neurons. These systems act across molecular, cellular, and network scales and exhibit context-dependent effects, making them difficult to model in real time (Dayan 2012; Tritsch et al. 2012; Boureau and Dayan 2010; Friston et al. 2013; Clark et al. 2018; Marder 2012). While neuroheuristics does not claim to resolve these intricacies, it offers a conceptual and methodological framework that supports the modeling of such multiscale, nonlinear interactions. This is achieved by integrating empirical insights and theoretical tools from systems neuroscience, such as adaptive gain theory, probabilistic reinforcement learning, and network dynamics modeling (Doya 2002; Cools and Robbins 2004; Villa 2008; Rutledge et al. 2014; de Berker et al. 2016; Grossberg 2013; Cabessa and Villa 2018; Shine 2019). Neuroheuristic methodologies thus facilitate the development of testable hypotheses concerning neuromodulatory function in both clinical and normative brain contexts, without overstating predictive precision.

The detection of recurrent neural patterns through advanced mathematical and statistical techniques is essential for addressing neurological and psychiatric conditions (Tetko and Villa 2001a; Aksenova et al. 2003; Asai et al. 2005; Guy et al. 2016; Masulli et al. 2020). By integrating computational modeling with experimental data, neuroheuristics offers a robust framework for investigating the interaction between high-order neural circuits and clinical pathologies, including Alzheimer’s disease (Celesia et al. 1993), Parkinson’s disease (Chibirova et al. 2005; Lintas et al. 2012), sleep disorders (Abe et al. 2021; Perrig et al. 2024), addiction (Synytsky et al. 2002; Spiga et al. 2011), epilepsy (Aksenova et al. 2007; Villa and Tetko 2010), and attention-deficit/hyperactivity disorder (ADHD) (Mesrobian et al. 2018; Dotare et al. 2020). In therapeutic domains, neuroheuristics supports the development of personalized, non-pharmacological interventions. For instance, targeted working memory training has shown promise for ADHD (Jaquerod et al. 2020; Dotare et al. 2021; Lintas et al. 2021), while cognitive behavioral therapy has proven effective for insomnia (Perrig et al. 2009).

## Conclusion

Despite substantial scientific progress, fundamental questions about the brain remain unresolved. How do large-scale neural networks integrate information across multiple sensory modalities to generate a unified experience? What are the precise neural correlates of consciousness? How can we improve our understanding and treatment of neurodegenerative and psychiatric disorders? These few questions underscore the extraordinary complexity of the brain and the persistent challenges it poses to researchers across disciplines. Unraveling the brain’s dynamic, multiscale, and temporally structured functions requires integrative strategies that bridge biology, computation, and cognition. Recent advancements in neuroscience, bolstered by technological innovation, are enabling researchers to make meaningful strides in deciphering the mechanisms underlying cognition, emotion, and self-awareness. In this context, neuroheuristics emerges as a promising transdisciplinary paradigm-one that promotes iterative hypothesis refinement, methodological flexibility, and the integration of diverse knowledge systems. By embracing the epistemological pluralism embodied in the neuroheuristic approach, contemporary neuroscience is positioned to move beyond static models and disciplinary silos. The convergence of emerging computational tools, molecular insights, and system-level models promises to accelerate our understanding of brain function and dysfunction. As this paradigm continues to evolve, it brings us closer to comprehending the core processes that underlie human thought, emotion, and behavior. thought and behavior.

## Data Availability

No datasets were generated or analysed during the current study.
